# Bilateral Simultaneous Quadriceps Tendon Rupture in a 24-Year-Old Obese Patient: A Case Report and Review of the Literature

**DOI:** 10.1155/2016/4713137

**Published:** 2016-10-20

**Authors:** Fahad H. Abduljabbar, Abdulaziz Aljurayyan, Bayan Ghalimah, Lawrence Lincoln

**Affiliations:** ^1^Division of Orthopedic Surgery, St. Mary's Hospital Center, McGill University, 3830 Lacombe Avenue, Montreal, QC, Canada H3T 1M5; ^2^Department of Orthopedic Surgery, King Abdulaziz University, Abdullah Sulayman St., Al Jamiah District, Jeddah 80200, Saudi Arabia; ^3^Department of Orthopedic Surgery, King Saud University, Riyadh 12372, Saudi Arabia

## Abstract

*Introduction*. Simultaneous bilateral quadriceps tendon ruptures (SBQTR) are uncommon knee injuries and most frequently occur in male patients, over 50 years of age. It can be associated with one or more predisposing risk factors like obesity, steroids use, and hyperparathyroidism. The main focus of this paper is to review SBQTR in obese patients.* Case Report*. We are reporting the youngest patient in the literature to date, a 24-year-old obese male patient, who presented to the emergency department complaining of bilateral knee pain and inability to walk after a fall during a basketball game. His clinical examination revealed the presence of a palpable suprapatellar gap and loss of knee extension bilaterally. Magnetic resonance imaging (MRI) confirmed that both of his quadriceps tendons were ruptured. A day after his diagnosis, the patient underwent successful operative repair followed by rehabilitation. At the two-year follow-up, the patient had full strength of both quadriceps muscles with no extension lag.* Conclusion*. The diagnosis of SBQTR can be challenging. Early diagnosis and treatment are associated with better functional outcome compared to delayed treatment. Physicians should have a high index of clinical suspicion in order not to miss such an injury and achieve favourable outcomes.

## 1. Introduction

Quadriceps tendon ruptures are not uncommon and occur typically in men older than 50 years old [1–4]. On the other hand, bilateral quadriceps tendons ruptures are rare [5]. Steiner and Palmer first described this entity in a 67-year-old man in 1949 [6]. However, some cases are reported in young patients with associated chronic illnesses [3, 7, 8]. Predisposing factors for quadriceps tendon rupture are numerous; these include diabetes mellitus, advanced age, obesity, chronic renal failure, hyperparathyroidism, systemic lupus erythematous, steroid use, gout, and pseudogout [9–13]. This report describes a 24-year-old male patient with simultaneous bilateral quadriceps tendons rupture (SBQTR) while playing basketball. To the best of our knowledge, this reports the youngest patient among other reports in the current literature.

## 2. Case Report

**Figure 1:**
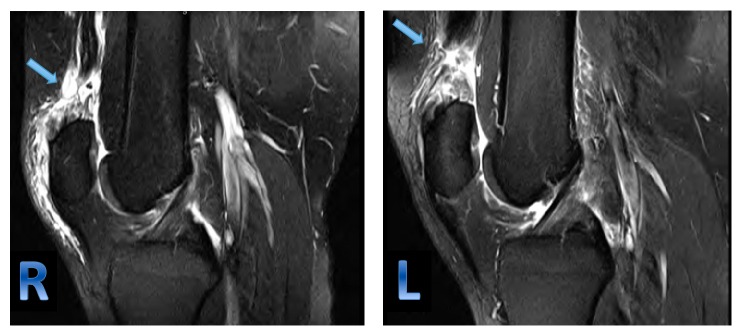
MRI of right and left knees showing T2-weighted sagittal image and demonstrating a full-thickness tear of the quadriceps tendon at the osteotendinous junction and fluid within the tendon gap with some retraction of the tendon which is more pronounced on the left side.

A 24-year-old male patient with a BMI of 35 kg/m^2^ (height 185 cm; weight 120 kg) who presented to the emergency department with severe bilateral knee pain and inability to walk. The mechanism of injury was sport-related. While playing basketball, the patient jumped and eccentrically loaded his left knee followed by a popping sound. While trying to maintain his balance by putting his weight on the contralateral leg, he heard a second popping sound, then experienced severe bilateral knee pain, and was not able to stand. His past medical history was unremarkable, and he had no history of steroid or fluoroquinolone intake. He is an active person and plays basketball on weekly basis. He had no history of prodromal knee pain prior to his injury. Clinical examination revealed a palpable gap in the suprapatellar region and loss of knee extension bilaterally. Magnetic resonance imaging (MRI) confirmed the diagnosis, which was bilateral quadriceps tendons rupture (Figure 1).

A day after his diagnosis with SBQTR, he was taken to the operating room for surgical repair. We approached both knees through standard anterior midline incisions. Intraoperatively, we identified both tears at the osteotendinous junction. We debrided all the frayed tissue and then repaired both tendons surgically using number 5 FiberWire® sutures (Arthrex, Naples, Florida) in a Whipstitch fashion. The free ends of the sutures were passed through three drilled holes in each patella using a 2.5 mm drill in a vertical orientation. These 3 holes were drilled using an anterior cruciate ligament (ACL) tibial tunnel guide which is used during this aspect of the procedure to manoeuvre the drill more accurately to the desired endpoint [21]. Using the tunnel guide decreases the risk of violating the articular surface, reduces the number of passes required to obtain an optimal position, minimizes injury to the patellar tendon, and eliminates the additional step of retrieving sutures through drill holes [21]. The drill is then replaced with a Beath pin. The inner limb of each suture is passed through the central tunnel, and then the outer limb is passed through the outer tunnels. All free limbs of the suture on both ends of the tendon were tied with the knee in full extension. We also found a complete retinacular tear in the left knee and only a small longitudinal split in the right knee and both were repaired using a number 1 absorbable suture.

**Figure 2:**
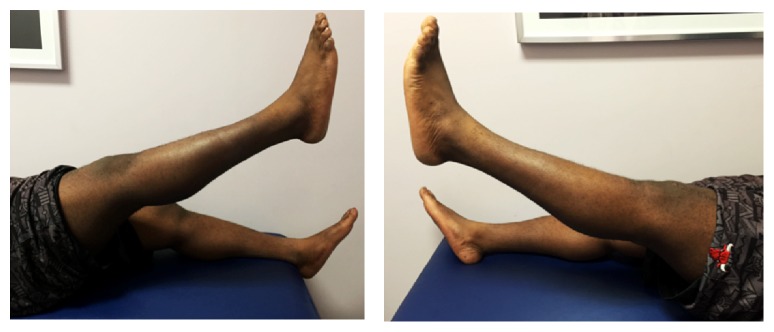
Clinical photos showing full active extension 2 years postoperatively without extension lag.

**Figure 3:**
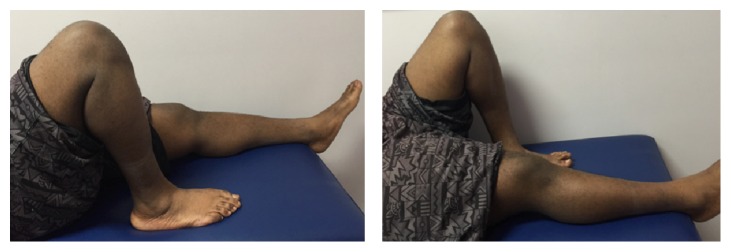
Clinical photos 2 years postoperatively showing full range of motion (0–120 degrees).

Postoperatively, both knees were protected in a knee immobilizer. The patient was allowed to bear weight as tolerated in full extension with the aid of crutches on both lower extremities. He started physiotherapy at 4 weeks to regain his knee range of motion (ROM) and quadriceps muscle strength. At the two-year follow-up, he had full quadriceps muscle strength with no extension lag (Figure 2), and his ROM was from 0 to 120 degrees bilaterally (Figure 3). At this point, the patient already resumed all his sport activities including playing basketball without limitations.

## 3. Discussion

Quadriceps tendon rupture (QTR) is not an uncommon occurrence and usually happens in male patients over 50 years old [2, 5, 8, 22]. The overall incidence of quadriceps tendon injuries is 1.37/100.000 [23]. They represent a big percentage of extensor mechanism injuries especially in patients older than 50 years old. Garner et al. reviewed extensor knee injuries over a 25-year period and found that 28.9% of them are quadriceps tendon ruptures [24]. Fortunately, simultaneous bilateral quadriceps tendons rupture (SBQTR) is rare and only 30% to 35% of them are spontaneous [19, 25]. Sometimes SBQTR can be associated with predisposing medical conditions including chronic renal disease, hyperparathyroidism, gout, systemic lupus erythematosus, diabetes mellitus, steroid use, obesity, and advanced age [8, 22, 26, 27]. In addition to that, there are other less common factors such as fluoroquinolones use, severe osteomalacia, and amyloidosis [22, 28]. Despite being associated with known predisposing risk factors, SBQTR can still occur in healthy individuals [8, 27].

In this paper, we are reporting the youngest patient in the literature with SBQRT secondary to a sport-related injury and obesity. The focus of this review will be on all reported cases of SBQRT in obese patients in the current literature. Obesity is one of the common risk factors for SBQRT. In a meta-analysis by Neubauer, obesity represented 10% of all reported risk factors for SBQRT [19]. With the modern sedentary life style and change in diet habits, obesity prevalence is on the rise. According to the National Health and Nutrition Examination Survey (NHANES), the obesity prevalence was relatively low and stable between 1960 and 1980 but more than doubled from 15% in 1980 to 34% in 2006 [29]. The WHO estimates that in 2005 approximately 1.6 billion people worldwide were overweight and that at least 400 million adults were obese. They further project that, by 2015, approximately 2.3 billion adults will be overweight and that at least 700 million will be obese [29]. These numbers are alarming as it could reflect the increased chance of having bilateral quadriceps tendon rupture in those obese patients. Yet there are no epidemiological studies to prove that.

Review of the English literature revealed slightly more than 100 cases of BQRT [9, 30]. Out of all reported patients, we identified 13 obese patients including the patient in this report with a mean age of 53 years (range, 24–75 years) (Table 1) [2, 3, 6, 14–20]. In the remaining 12 patients, the mechanism of injury was a mechanical fall in eleven patients and a spontaneous rupture while climbing stairs in one patient. Shah et al. showed in his review that these injuries happened spontaneously in patients with predisposing medical conditions while the remainder happened secondary to a significant eccentric loading of the tendons with the knee in a flexed position due to falling or participating in sports, more commonly basketball [8, 22, 25, 27, 31]. Six out of the 13 patients had other medical comorbidities in association with obesity. Apart from diagnostic difficulties, obesity itself may have a direct effect on the integrity of the tendon by causing fatty degeneration [7, 8, 28]. More importantly, the increased weight adds significant loading on the tendon, especially if eccentrically loaded (with a semiflexed knee) [18, 32].

Given the rare occurrence of these injuries, early diagnosis can present a challenge to the treating physician. Perfitt et al. reported that 67% of patients were misdiagnosed at their initial presentation [33]. The enlarged soft tissue envelope in obese patients can obscure the suprapatellar gap and make the diagnosis of QTR more challenging. Neubauer et al. reported 23 cases with delayed diagnosis of bilateral simultaneous rupture of the quadriceps tendon and they found that obesity was found most frequently among risk factors (21.4%) [19]. It is also common to misdiagnose ruptures in the elderly population. Strokes, occult fracture, rheumatoid arthritis, bilateral effusion, and other medical causes that may contribute to their inability to move their legs and therefore make it difficult to perform an appropriate extensor mechanism examination [18]. Failure to diagnose the injury from the initial presentation can delay the appropriate treatment and lead to a suboptimal clinical outcome [5, 27, 34]. To diagnose a quadriceps tendon rupture (QTR), the treating physician must obtain a thorough history and physical examination and supplement it with the appropriate imaging if required. Mechanism of the injury, predisposing medical conditions, and steroid use are key elements of the history. In the physical examination, the presence of knee pain, effusion, palpable suprapatellar gap, and extensor mechanism insufficiency can lead to the diagnosis [22, 26, 27]. Intact extensor mechanism can be misleading at times; this can happen in cases of QTR with intact medial and lateral retinacula; in these cases, further workup is required [35].

The knee extensor mechanism is evaluated while the patient is supine or sitting on the edge of the bed. The patient is asked to actively extend the knee. In some circumstances where the patient can not actively extend the knee, the physician can passively extend the knee lifting the heel off the bed and ask the patient to keep the knee extended. Failure to actively extend the knee or maintain a straight leg can indicate a dysfunction in the extensor mechanism. In the prone position, the knee can be flexed from a 90° position because of an intact hamstring mechanism, but extension will be impaired if the knee is flexed beyond 90° [36]. Further knee examination may show effusion and tenderness to palpation. The patella may be displaced and very mobile. A suprapatellar gap or depression may be palpated. The depression may be increased with active quadriceps muscle contraction. This is useful because an associated hemarthrosis can sometimes mask the suprapatellar gap [37].

Imaging can confirm the diagnosis if, in doubt, plain films, ultrasound (US), and MRI are valid and available options. Plain films are readily available in the emergency room and usually obtained routinely to rule out common differential diagnoses like fractures around the knee and particularly patellar fractures. Paying attention to subtle findings on the plain films like soft tissue defects and avulsed bone fragments at the very end of the quadriceps muscle, patella baja and knee effusion can facilitate the diagnosis of QTR [27, 38, 39]. Ultrasound has the advantages of being cheaper and easier to get compared to MRI; however, it is operator dependent and the diagnosis can be missed if performed by an inexperienced sonographer [20, 39]. MRI remains the modality of choice, as Perfitt et al. showed in his study that it has a 100% sensitivity and specificity with a positive predictive value of 100 in detecting a quadriceps tendon rupture compared to US [33, 35, 39], but increased cost and limited availability of MRI in the emergency setting are major limitations [20, 39].

The goal of the treating physician should be early diagnosis and surgical repair. Several studies suggested that early surgical repair and physiotherapy will result in superior outcomes compared to delayed repair [26, 27]. Emergency physicians, family physicians, and orthopedic surgeons should be familiar with diagnosing QTR from the initial presentation. Although very rare, SBQTR should be suspected even in young healthy patients. The educational value of this paper is to increase awareness of this entity among treating physicians, especially in obese patients in whom diagnosis might be difficult due to increased soft tissue envelope. Early diagnosis and early surgical management are crucial to achieve excellent outcome comparable to unilateral injuries.

## Figures and Tables

**Table 1:** Reported obese patients with SBQTR.

References	Age/sex	Mechanism of injury	Location of tear	Time before diagnosis	Risk factor(s)	BMI	Outcome
Steiner and Palmer, 1949 [6]	67/M	Slip and fall	NM	2 days	Obesity	NM	Ambulatory with AD after 5 weeks
Dalal and Whittam, 1966 [14]	63/M	Fall	OT	The same day	Obesity	NM	Extensor lag at 10 weeks
Firooznia et al., 1973 [15]	62/M	Fall	MT	NM	Obesity/DM	NM	NM
Julius, 1984 [3]	58/M	Fall	MT	The same day	Obesity	NM	Full ROM at 4 months
Dhar, 1988 [2]	75/M	Fall	MT	7 days	Obesity/HTN	NM	Extensor lag & ambulatory with AD at 4 months
	61/M	Fall	NM	2 days	Obesity	NM	Full ROM at 5 months
Nabors and Kremchek, 1995 [16]	43/M	Fall	OT & MT	2 weeks	Obesity	NM	Ambulatory with AD at 6 months
El-Zahaar, 1995 [17]	61/F	Fall	NM	The same day	Obesity/osteoporosis	NM	After 7 months, LT knee: 15 degrees of extension lag, RT knee: 20 degrees of extension lag, walks with a cane
Kelly et al., 2001 [18]	52/M	Fall	OT	The same day	Obesity	50.21	After 6 month, LT knee: 10 degrees of extension lag, RT knee: 25 degrees of extension lag
Neubauer et al., 2007 [19]	52/M	Fall	OT	4 weeks	Obesity/HTN	NM	Decreased ROM with good strength at 14 months
	30/M	Fall	OT	3 days	Obesity	NM	Full ROM & strength at 21 months
LaRocco et al., 2008 [20]	52/M	Walking up a flight of stairs	NM	2 days	Obesity/DM/HTN	NM	NM
Abduljabbar et al.	24/M	Sport injury	OT	The same day	Obesity	35	Full ROM & strength with no extension lag at 4 months. Back to sports at 1 year post-op

MT: musculotendinous junction, OT: osteotendinous junction, NM: not mentioned, AD: assistive device, ROM: range of motion, DM: diabetes mellitus, HTN: hypertension, LT: left, and RT: right.
